# Supplementary material for: EnzML: multi-label prediction of enzyme classes using InterPro signatures
Source: BMC Bioinformatics. 2012 Apr 25;13:61. doi: 10.1186/1471-2105-13-61 (PMC3483700; doi:10.1186/1471-2105-13-61)
Supplement: Addtional file 5 — The Java code to format the data files, evaluate and predict. The file enzml_java_code.tar.gz contains the Java code used to format database data to ARFF and XML formats, to execute cross and train-test (jackknife) evaluations and to record evaluation results to database. More information is included in the readme.txt file and the Javadoc files. The code can be used with a MySQL database. To use a different database software, other JDBC drivers might be required. [file 1471-2105-13-61-S5.gz › java_code/enzml2011/doc/uk/ac/ed/inf/csb/taweka2010/AttributeFactory.html]

AttributeFactory


---


|  |  |  |
| --- | --- | --- |
| **Package** \| **Class** \| **Use** \| **Tree** \| **Deprecated** \| **Index** \| **Help** | |  |
| **PREV CLASS**   **NEXT CLASS** | **FRAMES**    **NO FRAMES**     **All Classes** |  |
| SUMMARY: NESTED \| FIELD \| CONSTR \| METHOD | DETAIL: FIELD \| CONSTR \| METHOD |  |


---


## uk.ac.ed.inf.csb.taweka2010 Class AttributeFactory

```
java.lang.Object
  uk.ac.ed.inf.csb.taweka2010.AttributeFactory
```

---

``` public class AttributeFactory extends java.lang.Object ```

Attribute factory. Produces binary and nominal attributes.
A binary attribute is a special nominal attribute with only 2 options: yes/no (present/absent) (zero/one).
The attribute name often represents what is present or absent.
Example: if the attribute name is a protein domain, the value yes/no represents whether the domain is present or absent in that protein sequence (instance).
A nominal attribute is an attribute with a set list of possible string values.

**Version:**
:   20 Feb 2008

**Author:**
:   Luna De Ferrari luna.deferrari-at-ed.ac.uk

---

| **Field Summary** | |
| --- | --- |
| `static java.lang.String` | `ABSENT_OPTION` |
| `static java.lang.String` | `PRESENT_OPTION` |


**Constructor Summary**

`AttributeFactory()`


| **Method Summary** | |
| --- | --- |
| `static weka.core.Attribute` | `generateBinaryAttribute(java.lang.String attributeName)`             Generate a binary attribute (yes/no, present/absent) |
| `static weka.core.Attribute` | `generateNominalAttribute(java.lang.String attributeName, java.util.Vector<java.lang.String> values)`             Generate a nominal attribute (with a list of possible values) |
| `static weka.core.Attribute` | `generateNominalAttributeWithDummy(java.lang.String attributeName, java.util.Set<java.lang.String> classValues)`             Generate a nominal attribute (with a list of possible values) |
| `static weka.core.FastVector` | `getBinaryAttributeValuesSet()`             the options for a yes/no, present/absent attribute (beware: "no" has to be the first option for the sparse arff format to work) |

**Methods inherited from class java.lang.Object**

`equals, getClass, hashCode, notify, notifyAll, toString, wait, wait, wait`

**Field Detail**

### ABSENT\_OPTION

```
public static final java.lang.String ABSENT_OPTION
```

**See Also:**: Constant Field Values

---


### PRESENT\_OPTION

```
public static final java.lang.String PRESENT_OPTION
```

**See Also:**: Constant Field Values


**Constructor Detail**

### AttributeFactory

```
public AttributeFactory()
```


**Method Detail**

### getBinaryAttributeValuesSet

```
public static weka.core.FastVector getBinaryAttributeValuesSet()
```

:   the options for a yes/no, present/absent attribute
    (beware: "no" has to be the first option for the sparse arff format to work)

    :   **Returns:**: the vector with the two options

---


### generateBinaryAttribute

```
public static weka.core.Attribute generateBinaryAttribute(java.lang.String attributeName)
```

:   Generate a binary attribute (yes/no, present/absent)

    :   **Parameters:**: `attributeName` - the attribute name **Returns:**: the weka attribute

---


### generateNominalAttributeWithDummy

```
public static weka.core.Attribute generateNominalAttributeWithDummy(java.lang.String attributeName,
                                                                    java.util.Set<java.lang.String> classValues)
```

:   Generate a nominal attribute (with a list of possible values)

    :   **Parameters:**: `attributeName` - the attribute name **Returns:**: the weka attribute

---


### generateNominalAttribute

```
public static weka.core.Attribute generateNominalAttribute(java.lang.String attributeName,
                                                           java.util.Vector<java.lang.String> values)
```

:   Generate a nominal attribute (with a list of possible values)

    :   **Parameters:**: `attributeName` - the attribute name **Returns:**: the weka attribute


---


|  |  |  |
| --- | --- | --- |
| **Package** \| **Class** \| **Use** \| **Tree** \| **Deprecated** \| **Index** \| **Help** | |  |
| **PREV CLASS**   **NEXT CLASS** | **FRAMES**    **NO FRAMES**     **All Classes** |  |
| SUMMARY: NESTED \| FIELD \| CONSTR \| METHOD | DETAIL: FIELD \| CONSTR \| METHOD |  |


---
